# Growth Hormone Deficiency and Systemic Metabolic Remodeling: Insights from Metabolomics and Lipidomics

**DOI:** 10.3390/metabo16100744

**Published:** 2026-10-02

**Authors:** Sarmed Al-Samerria, Sally Radovick

**Affiliations:** Department of Pediatrics, University of Arizona College of Medicine—Tucson, Tucson, AZ 85724, USA; sradovick@arizona.edu

**Keywords:** growth hormone deficiency, growth hormone bioactivity, metabolomics, lipidomics, biomarker discovery, metabolic profiling, growth hormone replacement

## Abstract

Growth hormone (GH) deficiency (GHD) is a systemic endocrine–metabolic disorder affecting growth, body composition, substrate handling, and cardiometabolic health. Clinical evaluation relies primarily on GH stimulation testing and circulating insulin-like growth factor 1 (IGF-1), but these measures incompletely reflect downstream tissue-level consequences of impaired GH bioactivity. Metabolomic and lipidomic profiling provide systems-level approaches for characterizing these consequences. Evidence from experimental models and human studies implicates coordinated alterations in amino-acid turnover, mitochondrial and central energy metabolism, fatty-acid handling, and lipid remodeling, consistent with disrupted substrate exchange across GH-responsive tissues. These signatures are influenced by sex, developmental stage, adiposity, nutritional state, treatment exposure, and analytical platform, limiting the interpretation of individual metabolites as universal biomarkers. In this review, we integrate current metabolomic and lipidomic evidence with established GH biology, including temporal and sex-dependent signaling, tissue-specific metabolic actions, and responses to GH replacement. We further evaluate the potential of metabolic signatures for biological phenotyping, treatment-response assessment, and pharmacodynamic monitoring. Current evidence supports metabolomics and lipidomics as promising functional readouts of altered GH action, but not yet as validated diagnostic tools. Progress toward clinical translation will require standardized study designs, longitudinal sampling, sex- and development-aware analyses, harmonized analytical workflows, and independent validation in well-characterized cohorts.

## 1. Introduction

Growth hormone (GH) deficiency (GHD) is commonly recognized through impaired linear growth in childhood or reduced somatotropic activity in adulthood, but its consequences extend well beyond growth. GH contributes to the regulation of body composition, protein turnover, lipid mobilization, substrate partitioning, and broader cardiometabolic physiology. In children, these actions occur during rapid growth and pubertal maturation; in adults, untreated GHD is associated with increased adiposity, reduced lean mass, and impaired physical and metabolic function. GHD is therefore better considered a systemic endocrine–metabolic disorder than a condition defined solely by stature, circulating GH, or insulin-like growth factor 1 (IGF-1) concentrations [1,2,3,4,5].

Clinical evaluation of GHD relies on auxology, clinical history, pituitary imaging when appropriate, GH stimulation testing, and circulating IGF-1 concentrations. These approaches remain central to diagnosis and management, but each has limitations as a measure of downstream GH action. Random GH concentrations have little diagnostic value because secretion is episodic and pulsatile, while stimulation-test responses vary with developmental stage, adiposity, assay methodology, and diagnostic thresholds. IGF-1 provides a more stable index of GH-dependent activity, but a normal concentration does not exclude GHD, while a low concentration is not specific and may reflect undernutrition, liver dysfunction, chronic illness, or other physiological factors. Conventional endocrine testing can therefore identify impaired somatotropic function without fully defining the metabolic state of GH-responsive tissues [1,4,5,6,7].

This distinction is important because GH action depends on both hormone exposure and biological context. Signaling through the growth hormone receptor (GHR), Janus kinase 2 (JAK2), and signal transducer and activator of transcription 5 (STAT5) regulates endocrine IGF-1 production and metabolic programs in the liver and other GH-responsive tissues. The temporal organization of GH secretion, particularly sex-dependent differences in pulse structure and hepatic STAT5 activation, further modifies downstream transcriptional and metabolic responses. Metabolomic and lipidomic profiling may therefore complement conventional endocrine measurements by capturing integrated biochemical consequences of altered GH bioactivity across tissues. Emerging experimental and clinical studies repeatedly implicate amino-acid metabolism, mitochondrial substrate utilization, and lipid remodeling, although these signatures remain strongly dependent on sex, developmental stage, treatment exposure, and metabolic context [8,9,10,11,12].

This narrative review, supported by a structured literature search, evaluates metabolomic and lipidomic evidence for GHD as a systemic metabolic disorder rather than simply cataloguing altered metabolites and lipids. We address three questions: (1) which pathway-level alterations recur across human and experimental studies; (2) how sex, developmental stage, body composition, disease context, and GH replacement modify these signatures; and (3) whether current evidence supports diagnostic, phenotypic, pharmacodynamic, predictive, or metabolic-risk applications and what validation is required for translation. We integrate direct human GHD evidence with experimental and mechanistic studies of altered GH signaling, while distinguishing their relative evidentiary relevance.

## 2. Literature Search and Review Methodology

This review was conducted as a narrative synthesis supported by a structured literature search. PubMed/MEDLINE, Scopus, and Web of Science were searched through August 2026 using combinations of terms related to “growth hormone deficiency,” “growth hormone,” “GH replacement,” “growth hormone receptor,” “GH resistance,” “metabolomics,” “metabolome,” “lipidomics,” “lipidome,” “metabolic profiling,” “biomarker,” “treatment response,” “sex differences,” “pediatric,” “adult,” and “development.” Reference lists of relevant primary studies and reviews were also examined to identify additional publications of direct relevance.

Priority was given to peer-reviewed human and experimental studies reporting metabolomic or lipidomic measurements in GHD, reduced GH secretion, GH replacement, or altered GH signaling. Human studies of classical GHD were treated as the most direct evidence. Human studies of altered GH signaling outside classical GHD, experimental models of reduced GH action, GH resistance, and tissue-specific disruption of GH signaling were included when they provided mechanistic context for interpreting metabolic pathways relevant to GHD.

Because the available literature is heterogeneous with respect to population characteristics, developmental stage, sex distribution, GHD etiology, treatment exposure, sample type, analytical platform, preprocessing strategy, statistical analysis, and reported endpoints, quantitative meta-analysis was not considered appropriate. Evidence was therefore synthesized narratively, with emphasis on recurrent pathway-level findings, direction of reported metabolic changes, biological context, methodological heterogeneity, and potential relevance to biomarker development.

This review was not designed as a systematic review, and no formal risk-of-bias scoring or quantitative evidence grading was performed. The synthesis should therefore be interpreted as a structured narrative evaluation of the current evidence rather than an exhaustive systematic assessment.

## 3. GH-IGF-1 Signaling and Metabolic Regulation

**Figure 1:**
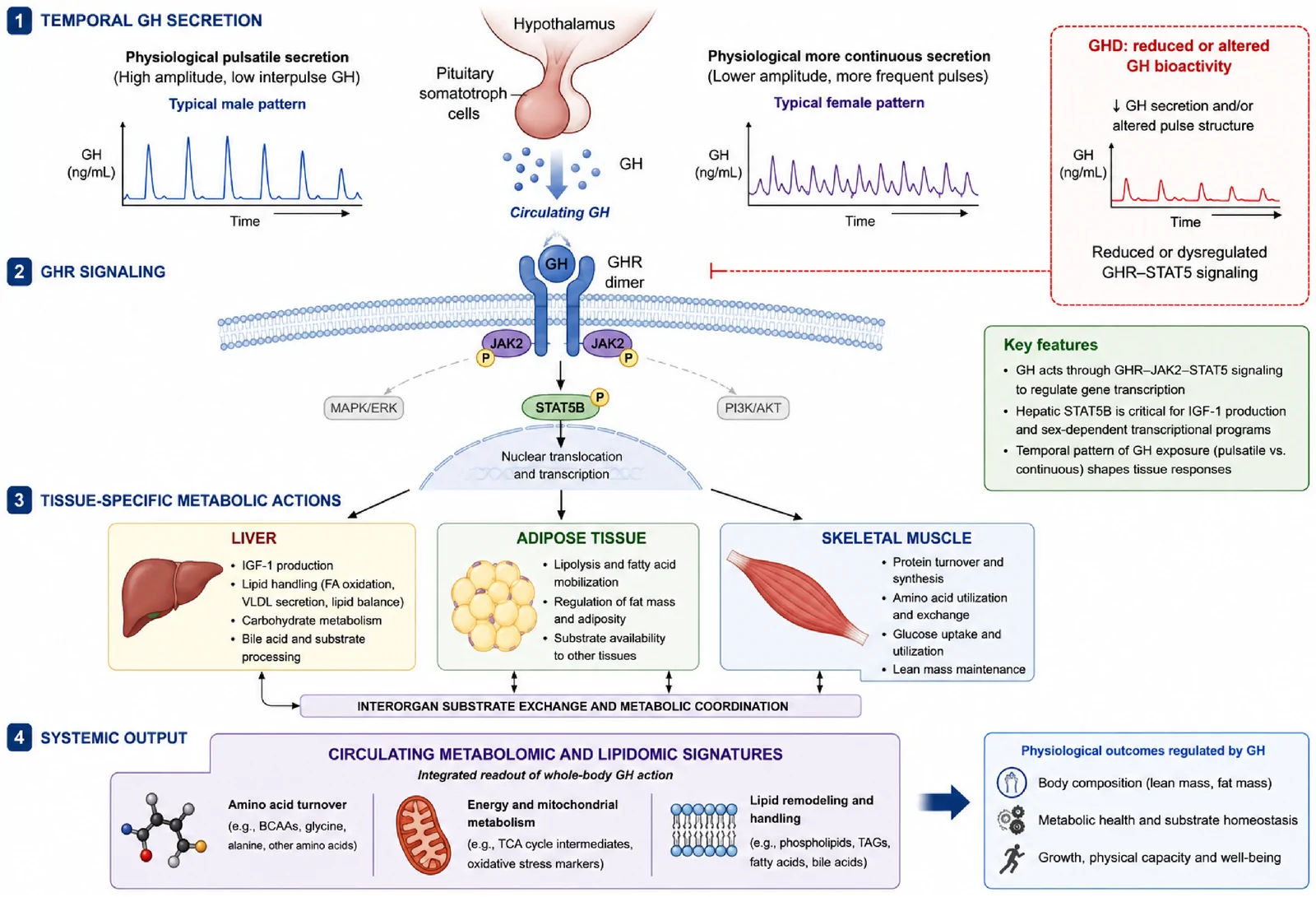
Temporal and tissue-specific regulation of metabolism by growth hormone. This schematic provides the mechanistic framework used to interpret the omics evidence reviewed in the text. Endogenous GH secretion is temporally organized and sex-dependent; GHR-JAK2-STAT5 signaling links pituitary GH exposure to hepatic IGF-1 production and transcriptional regulation, while coordinated actions in liver, adipose tissue, and skeletal muscle influence lipid mobilization, glucose and energy metabolism, and protein/amino-acid turnover. The key message is that circulating metabolomic and lipidomic signatures can reflect both direct GH-dependent tissue regulation and secondary consequences of altered body composition and substrate flux. Solid arrows indicate the primary directional relationships depicted; dashed lines indicate secondary or reduced/inhibitory signaling relationships. P denotes phosphorylation. GH, growth hormone; GHD, growth hormone deficiency; IGF-1, insulin-like growth factor 1.

The interpretation of metabolomic and lipidomic signatures in GHD requires a mechanistic framework linking pituitary GH secretion to tissue metabolism (Figure 1). GH binding to GHR activates JAK2-STAT5 signaling and related pathways that coordinate growth and metabolic regulation. Hepatic STAT5B is central to endocrine IGF-1 production and GH-dependent transcriptional programs, including pathways that influence lipid handling, carbohydrate metabolism, amino-acid and nitrogen metabolism, and bile-acid regulation. In parallel, direct GH actions in adipose tissue and skeletal muscle influence lipolysis, substrate availability, protein turnover, and insulin-sensitive fuel use. Additional MAPK/ERK and PI3K/AKT signaling contributes to tissue-specific responses. Thus, disruption at the level of GH exposure, GHR-JAK2-STAT5 signaling, or tissue responsiveness can generate metabolomic and lipidomic changes through both direct tissue effects and secondary alterations in body composition and substrate flux [10,13,14,15,16,17].

The metabolic consequences of altered GH action are further modified by biological context. Sex and secretory pattern influence hepatic STAT5 activation and sex-biased metabolic transcription; developmental stage changes anabolic demand and substrate allocation; nutritional state and adiposity alter insulin sensitivity and lipid flux; and tissue responsiveness determines how a given GH exposure is translated into metabolic output. These modifiers help explain why similar endocrine abnormalities can yield different circulating metabolomic and lipidomic profiles and why such profiles should be interpreted as context-dependent functional readouts rather than isolated biochemical abnormalities [11,18,19].

### 3.1. GH Pulsatility and Temporal Control of Metabolic Signaling

GH action depends on both hormone abundance and temporal exposure. Endogenous GH secretion is episodic, and pulse amplitude, frequency, and interpulse concentrations vary with sleep, nutrition, adiposity, sex steroids, age, and developmental stage. Repeated cycles of receptor activation, STAT5 signaling, transcriptional induction, and signal termination therefore convey information that is not captured by a single GH or IGF-1 measurement [5,11,13,19,20].

The liver provides the clearest example of this temporal regulation. High-amplitude GH pulses separated by low interpulse levels, typical of the male pattern, generate a different hepatic STAT5 and transcriptional program from the more continuous female pattern. These sex-dependent signaling states influence pathways involved in lipid handling, carbohydrate metabolism, bile-acid regulation, and broader substrate processing [11,18,19].

This temporal biology is relevant to metabolomic and lipidomic studies because reduced secretion, altered pulse structure, and nonphysiological replacement patterns may produce different downstream metabolic signatures. Daily and long-acting GH therapies improve endocrine and clinical outcomes but do not reproduce endogenous pulsatile secretion; metabolomic findings during treatment should therefore be interpreted in relation to formulation, exposure pattern, sampling time, sex, and tissue responsiveness.

### 3.2. Tissue-Specific Metabolic Actions of GH

GH regulates systemic metabolism through coordinated actions across the liver, adipose tissue, and skeletal muscle (Figure 1). In the liver, GH supports endocrine IGF-1 production and regulates substrate handling, lipid balance, and energy metabolism; disruption of hepatic GH signaling promotes lipid accumulation and altered fatty-acid metabolism [10,21,22]. These hepatic effects are especially relevant because the liver integrates GH input with nutritional state, insulin signaling, and circulating substrate availability.

Adipose tissue is a major target of GH action. GH promotes lipid mobilization and regulates adipose distribution and metabolic function, particularly during fasting or increased energy demand [23,24]. Reduced GH action can diminish lipolytic drive, increase adiposity, and alter circulating fatty-acid availability. Importantly, these body-composition changes can themselves reshape the metabolome through secondary effects on insulin sensitivity, hepatic lipid flux, and substrate partitioning; not every circulating lipid change should therefore be interpreted as a direct consequence of GHR signaling [23,24,25].

Skeletal muscle contributes to protein turnover, amino-acid exchange, insulin-sensitive glucose handling, and whole-body energy expenditure. Reduced GH bioactivity is associated with lower lean mass and impaired anabolic balance, which may alter circulating amino acids and energy-related metabolites [24,26,27]. Together, liver, adipose tissue, and skeletal muscle provide the tissue framework summarized in Figure 1: circulating metabolic signatures in GHD likely integrate direct GH-dependent regulation with secondary consequences of altered adiposity, lean mass, insulin sensitivity, and interorgan substrate flux.

### 3.3. Sex Dimorphism and Developmental Context

Sex and developmental stage strongly influence GH action and the metabolic phenotype of GHD. During childhood and adolescence, metabolomic signals are superimposed on growth acceleration, pubertal maturation, changing body composition, and shifting substrate demand. Pediatric signatures should therefore be interpreted against age- and puberty-dependent physiology rather than attributed solely to reduced GH secretion [1,2,5].

Sex-specific GH secretory patterns and hepatic STAT5 activation further shape lipid, carbohydrate, and bile-acid metabolism. Consequently, males and females may show different metabolic responses to GHD and GH replacement, and pooled-sex analyses can obscure biologically meaningful effects. These principles are revisited in Section 5 in the context of the available metabolomic evidence [11,18,19,28].

## 4. Metabolomic and Lipidomic Signatures of Altered GH Bioactivity

**Figure 2:**
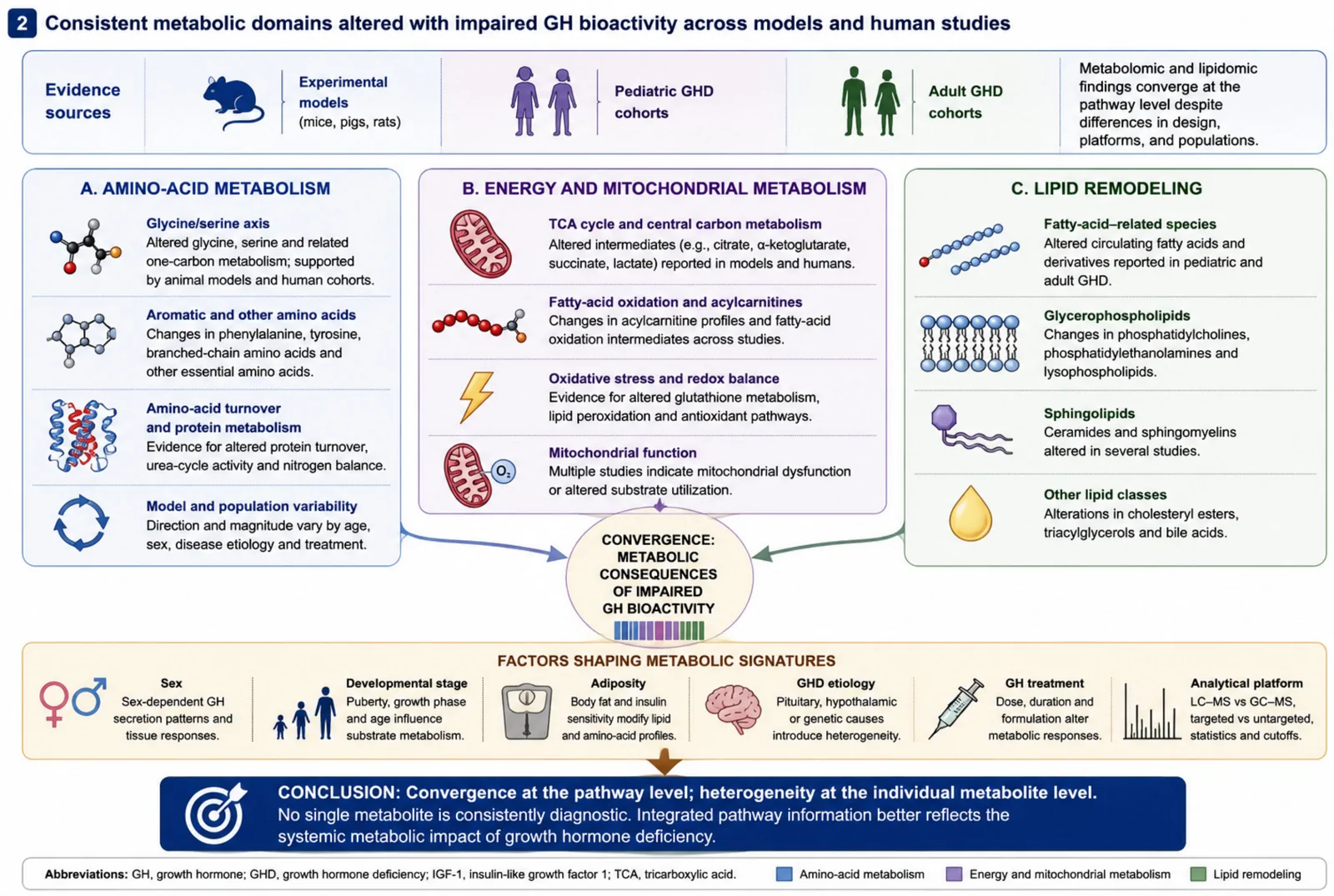
Convergent metabolic domains associated with impaired growth hormone bioactivity across experimental and human studies. The figure synthesizes the primary evidence reviewed in the text and the mechanistic studies discussed in Section 3, Section 4 and Section 5. Convergence is strongest at the pathway level, particularly for amino-acid metabolism, energy and mitochondrial substrate utilization, fatty-acid handling, and lipid remodeling. Individual metabolite and lipid changes vary with sex, developmental stage, adiposity, GHD etiology, GH treatment, sampling conditions, and analytical platform; the figure therefore emphasizes recurrent biological domains rather than a universal diagnostic signature. Colors denote the pathway domains shown in the schematic: blue, amino-acid metabolism; purple, energy and mitochondrial metabolism; green, lipid remodeling. Colors do not indicate the direction or magnitude of change.

Metabolomic and lipidomic studies provide downstream biochemical readouts of impaired GH bioactivity. Throughout this section, evidence is interpreted according to its proximity to classical GHD: direct human GHD studies are given greatest clinical weight, while human studies of altered GH signaling outside classical GHD, experimental models of reduced GH action, and mechanistic GH/GHR models are used to support biological interpretation. Across these evidence types, the most recurrent domains involve amino-acid metabolism, energy and mitochondrial substrate utilization, fatty-acid handling, and lipid remodeling (Figure 2). Individual metabolites are less reproducible, in part because sex, developmental stage, body composition, treatment exposure, sampling conditions, and analytical platform modify the observed profile [8,9,12,29,30].

### 4.1. Amino-Acid Metabolism and Nitrogen Balance

Amino-acid metabolism is recurrently implicated in GHD, consistent with the role of GH in protein turnover, nitrogen balance, and muscle–liver substrate exchange. Direct human and experimental studies report changes in amino acids and related metabolites, although the specific compounds and directions vary across populations and platforms [8,9,12,29,30]. The most defensible interpretation is therefore pathway-level: altered amino-acid signatures may reflect reduced anabolic signaling, lower lean tissue mass, altered proteolytic release, and changes in hepatic substrate routing. These mechanisms also illustrate an important distinction between direct GH effects and metabolomic changes mediated secondarily through body composition and substrate demand.

Skeletal muscle is a major reservoir of circulating amino acids and a key site through which GH influences protein metabolism and lean tissue maintenance. GH acts through both IGF-1-dependent and direct GHR-mediated mechanisms to support anabolic balance, amino-acid utilization, and preservation of muscle metabolic capacity. When GH bioactivity is reduced, this balance may weaken, limiting amino-acid incorporation into structural and metabolic proteins and increasing reliance on proteolytic release during periods of metabolic demand. These changes may contribute to altered circulating amino-acid profiles in GHD, particularly when reduced lean mass or impaired physical capacity is present. Therefore, amino-acid changes detected by metabolomic profiling should not be dismissed as dietary variation or nonspecific metabolic noise; they may also reflect impaired muscle anabolic signaling and altered amino-acid exchange between muscle and the circulation [24,26,27].

The liver provides another major site through which altered GH bioactivity may shape circulating amino-acid signatures. Amino acids released from skeletal muscle are routed through hepatic pathways that determine whether their carbon skeletons support protein synthesis, gluconeogenesis, oxidative metabolism, or nitrogen disposal. GH signaling contributes to this coordination through hepatic IGF-1 production and transcriptional regulation of metabolic pathways involved in substrate handling. In GHD, reduced hepatic GH input may weaken this regulatory control, allowing amino-acid substrates to be redirected away from anabolic retention and toward glucose production, oxidation, or ureagenesis. This provides a plausible mechanistic link between impaired GH signaling, altered liver–muscle substrate exchange, and the amino-acid changes reported in metabolomic studies of GHD. The key point is not that any single amino acid defines GH deficiency, but that coordinated amino-acid shifts may reflect altered hepatic routing of substrates under reduced GH bioactivity [9,10,13,31,32,33].

Taken together, amino-acid findings are more reproducible at the pathway level than at the level of individual metabolites. Their expression is modified by developmental stage, sex, adiposity, nutritional state, disease severity, and treatment exposure, which limits the reliability of any single amino acid as a universal GHD biomarker [8,12,29,30,33].

### 4.2. Mitochondrial Substrate Utilization and Energy Metabolism

Energy and mitochondrial substrate metabolism represent another recurrent domain in GHD-related profiling. Studies have reported changes in TCA-cycle intermediates, carbohydrate-related metabolites, acylcarnitines, oxidative-stress markers, and related pathways, although the individual signals vary across models and cohorts [9,34,35,36,37,38]. These findings should be interpreted as evidence of altered fuel selection, oxidative substrate handling, or metabolic flexibility rather than as proof of primary mitochondrial dysfunction. Secondary effects of adiposity, reduced lean mass, and altered substrate delivery may contribute substantially to these signatures.

The liver is a central site where GH-dependent signaling influences mitochondrial substrate handling. Rather than simply regulating mitochondrial abundance, hepatic GH signaling appears to support oxidative respiratory function, ATP production, redox balance, and the coordinated use of lipid- and carbohydrate-derived substrates. Experimental disruption of hepatic GHR signaling has been associated with impaired oxidative phosphorylation, reduced ATP availability, increased reactive oxygen species production, and altered expression of genes involved in fatty-acid uptake, lipid synthesis, β-oxidation, and central carbon metabolism. These findings suggest that intact hepatic GH signaling helps preserve mitochondrial respiratory capacity and metabolic flexibility, particularly under conditions in which fasting, nutrient availability, or adipose-derived fatty-acid flux impose increased oxidative demand [10,35,36,37].

Reduced GH bioactivity may therefore disrupt energy metabolism by weakening the coordination between substrate availability and oxidative utilization. In GHD, impaired adipose lipid mobilization, altered hepatic fatty-acid oxidation, and reduced lean mass may collectively shift fuel handling toward lipid storage, compensatory carbohydrate use, or greater reliance on amino-acid-derived carbon skeletons. This does not imply a uniform collapse of mitochondrial function. A more plausible interpretation is that GHD alters fuel selection and reduces the efficiency with which liver and peripheral tissues coordinate lipid, glucose, and amino-acid substrates for mitochondrial oxidation. This framework is consistent with metabolomic and proteomic evidence linking impaired GH signaling to changes in TCA cycle-related metabolites, amino-acid degradation pathways, urea cycle-associated enzymes, and oxidative-stress markers [9,37,38].

The systemic consequences of altered fuel handling are relevant to circulating metabolite profiles, but interorgan substrate coordination is generally inferred rather than directly measured in human GHD studies. A cautious interpretation is that reduced GH action may alter the relationship between adipose fatty-acid release, hepatic and peripheral substrate use, and compensatory carbohydrate or amino-acid metabolism. Mitochondrial-related signatures should therefore be framed as integrated markers of altered fuel partitioning and oxidative metabolism, not as direct evidence of intrinsic mitochondrial failure.

### 4.3. Lipid Mobilization, Fatty-Acid Metabolism, and Lipid Remodeling

GH is an important regulator of lipid mobilization, particularly through its actions on adipose tissue during fasting and other energy-demanding states. By promoting lipolysis, GH increases the availability of circulating free fatty acids for hepatic and peripheral oxidative metabolism, thereby coordinating adipose substrate release with systemic energy requirements [24,39]. These effects involve both acute regulation of adipocyte lipolysis and longer-term changes in lipid-storage and lipid-droplet pathways, providing a mechanistic basis for the altered fatty-acid and lipid profiles observed when GH action is impaired or restored [38,40,41].

In GHD, reduced GH-dependent lipid mobilization may contribute to increased fat mass, altered fatty-acid availability, and impaired coupling between adipose lipid release and downstream substrate use. These changes provide a mechanistic basis for interpreting lipidomic alterations in GHD as markers of disrupted lipid trafficking rather than isolated changes in lipid abundance. This distinction is important because circulating lipid species may reflect not only adipose storage or release, but also hepatic uptake, mitochondrial fatty-acid use, and remodeling of structural lipid pools.

The liver is a major site at which altered GH-dependent substrate flux is translated into metabolic phenotype. Intact hepatic GH signaling contributes to the regulation of fatty-acid uptake, de novo lipogenesis, triglyceride turnover, and oxidative lipid disposal, whereas disruption of hepatic GHR signaling in experimental models promotes hepatic lipid accumulation and metabolic dysfunction [10,42]. Consistent with these experimental findings, lower activity of the GH/IGF-1 axis in humans has been associated with greater intrahepatic lipid accumulation and markers of hepatocellular injury [21]. Interventional studies further support a relationship between GH activity and hepatic lipid handling. Recombinant human GH administration reduced intrahepatic lipid content in adults with overweight/obesity and nonalcoholic fatty liver disease, while tesamorelin, a growth hormone-releasing hormone analogue that stimulates endogenous GH secretion, reduced liver fat in people with HIV and abdominal adiposity [43,44]. Together, these findings support a model in which reduced GH bioactivity favors hepatic lipid accumulation, whereas augmentation of GH signaling can promote a more favorable hepatic lipid phenotype in selected metabolic contexts.

Lipidomic profiling provides evidence that impaired GH bioactivity is associated with remodeling of circulating lipid species beyond conventional measures such as total triglycerides and cholesterol. Recent studies in both pediatric and adult GHD have identified alterations across multiple lipid species and lipid classes, although the magnitude and direction of these changes are not uniform across populations. In children with GHD, untargeted serum metabolomics identified prominent lipid-related differences, including lipid clusters associated with GHD, whereas high-coverage targeted lipidomics in adults demonstrated a broader pattern of serum lipid dysregulation [12,45]. Together, these findings support lipid remodeling as a component of the systemic metabolic phenotype of GHD, while also emphasizing that the specific lipidomic signature is likely influenced by age, sex, adiposity, treatment status, and analytical methodology. Lipidomic profiling may therefore provide complementary information on altered lipid handling that is not captured by conventional circulating lipid measurements.

Lipidomic changes in GHD are best interpreted as pathway-level evidence of disrupted lipid trafficking, storage, and remodeling rather than as isolated abnormalities in individual lipid species. Reduced GH bioactivity may weaken adipose lipid mobilization, alter hepatic lipid disposal, increase lipid storage, and modify membrane lipid composition. A pathway-level interpretation is therefore more defensible than reliance on any single lipid marker. Within this framework, lipidomic profiling may help define how impaired GH signaling reshapes adipose-liver communication, hepatic lipid burden, and fatty-acid use, while identifying candidate lipid signatures for validation in longitudinal and treatment-response studies [9,21,24,45].

### 4.4. Integrated Metabolomic Patterns Across Animal and Human Studies

Across animal models and human cohorts, metabolomic and lipidomic studies point more consistently to recurrent pathway domains than to a single disease-specific metabolite signature (Figure 2). The strongest convergence involves amino-acid metabolism, energy and mitochondrial substrate utilization, fatty-acid handling, and lipid remodeling [8,9,12,30,37,45,46,47]. The evidentiary context remains important: direct human GHD studies provide the most clinically relevant observations, whereas experimental GHD, GH-resistance, and tissue-specific GHR models are primarily mechanistic. Pediatric findings are additionally shaped by growth and pubertal substrate demand, while adult profiles are more strongly influenced by chronic adiposity, lean mass, insulin sensitivity, treatment history, and cardiometabolic context.

Differences across studies are therefore not automatically contradictory. The major sources of variability can be considered hierarchically: biological factors (age/developmental stage, sex, adiposity, GHD etiology and severity), treatment factors (GH exposure, formulation, dose and duration), pre-analytical factors (fasting, sampling time, recent food intake or exercise, specimen processing), analytical factors (platform coverage and annotation), and statistical factors (normalization, multiple-testing correction and feature selection). Animal models control many of these variables but have limited clinical generalizability, whereas human studies offer direct relevance at the cost of greater biological heterogeneity [8,9,37,45].

Current evidence therefore supports metabolomic and lipidomic profiling as a pathway-level readout of impaired GH bioactivity rather than a fixed biomarker panel. Figure 2 summarizes this convergence and the principal modifiers that shape individual signatures. These modifiers should be incorporated into study design and interpretation rather than treated simply as statistical nuisance factors.

## 5. Sex-Specific and Developmental Dimensions of GHD Metabolomics

Sex and developmental stage are not minor covariates in GHD metabolomics; they are core biological variables that shape how GH deficiency is expressed in circulating metabolite and lipid profiles. This is particularly important because GH action differs by secretory pattern, tissue responsiveness, and body-composition context, all of which can influence downstream metabolic readouts. In pediatric GHD, metabolomic signatures are superimposed on growth, pubertal maturation, and changing energy demands, making it difficult to separate disease-related effects from normal developmental metabolism. These factors do not simply confound interpretation; they modify the biological consequences of reduced GH bioactivity. For this reason, sex-specific and developmental context should be built into study design, statistical analysis, and biomarker interpretation rather than treated as secondary adjustments after metabolomic data are generated [1,8,11].

### 5.1. Sex-Specific Metabolomic Responses to Altered GH Bioactivity

Evidence for sex-specific metabolomic responses remains limited but supports sex-aware analysis. Experimental studies show that reduced GH bioactivity and GH treatment can produce different metabolic responses in males and females, with some metabolites appearing GH-responsive in one sex but not the other [9]. Human studies are generally underpowered for formal sex-stratified analysis, so current evidence supports sex as a core design variable rather than a validated sex-specific diagnostic framework [8,45].

Evidence for sex-specific metabolomic responses in GHD remains limited, but available studies support the need for sex-aware interpretation. In experimental models, sex-stratified metabolomic profiling has shown that reduced GH bioactivity and GH treatment do not produce identical metabolic responses in males and females. Some metabolites appear GH-responsive in one sex but not the other, while others remain altered despite treatment, suggesting that circulating signatures may include both GH-dependent and GH-independent components. This distinction matters because pooled analyses can obscure sexually dimorphic responses and present mixed-sex averages as if they were universal biomarkers. Human pediatric and adult studies have begun to identify metabolomic and lipidomic differences in GHD, but many remain constrained by small cohort size, uneven sex distribution, and limited power for formal sex-stratified analysis. Current evidence therefore supports sex-specific metabolomics as a necessary design principle, not yet as a validated clinical framework [8,45].

### 5.2. Pediatric Versus Adult GHD

Pediatric GHD differs from adult GHD because its metabolic phenotype is superimposed on active growth, tissue accretion, pubertal maturation, changing insulin sensitivity, and evolving sex-steroid exposure. Amino-acid and lipid signatures in children may therefore reflect an interaction between reduced GH-dependent anabolic signaling and growth-related substrate demand rather than a fixed biochemical marker of deficiency [1,2,8]. Age- and puberty-aware interpretation is essential before pediatric signatures can be compared across cohorts or extrapolated to adults.

In adults, linear growth and pubertal maturation are complete, so metabolomic and lipidomic profiles are less dominated by developmental substrate demand and more strongly shaped by visceral adiposity, lean mass, insulin sensitivity, physical activity, aging, pituitary disease context, and hormone replacement. Direct adult GHD studies include the fasting serum GC-MS study of Höybye et al. (10 GHD and 10 matched controls), the untargeted UPLC-MS study of Yang et al. (31 GHD and 31 controls), and the targeted lipidomics study of Yang et al. (30 GHD and 30 BMI-matched controls) [30,46,48]. Together, these studies emphasize lipid-related, fatty-acid, amino-acid, and energy-metabolic alterations, while also illustrating how cohort etiology, adiposity, platform, and treatment exposure shape the observed profile.

The distinction between pediatric and adult GHD is therefore not simply a matter of age group, but of biological context. In children, metabolomic signatures are shaped by the interaction between impaired GH bioactivity and growth-related substrate demand. In adults, the same pathway domains may reflect longer-term changes in adiposity, lean mass, lipid handling, insulin sensitivity, and chronic substrate partitioning. This means that amino-acid, mitochondrial-related, and lipid signatures should not be assumed to have identical biological meaning across pediatric and adult cohorts. A metabolite linked to growth failure in childhood may reflect reduced anabolic substrate use, whereas a similar change in adults may reflect altered body composition or cardiometabolic risk. Future studies should therefore avoid combining pediatric and adult GHD as biologically interchangeable groups and should instead stratify analyses by developmental stage, sex, adiposity, treatment exposure, and pituitary disease context.

### 5.3. Treatment Exposure and GH Replacement

GH replacement changes the biological baseline against which metabolomic and lipidomic signatures are interpreted. In children with GHD, treatment increases growth velocity and modifies IGF-1 production, lean mass accrual, fat mass, and energy requirements. Post-treatment metabolite profiles may therefore reflect both correction of impaired GH bioactivity and the metabolic demands of accelerated growth. In adults, GH replacement is used to address the broader clinical phenotype of GHD, including altered body composition, reduced physical capacity, adverse lipid profiles, and impaired quality of life, but its metabolic effects vary with age, adiposity, dose titration, treatment duration, pituitary disease context, and baseline cardiometabolic risk. Recent work also highlights that treatment-related metabolic changes may differ between children with GHD and other growth disorders, reinforcing the need to interpret GH exposure, diagnostic category, and treatment duration together rather than treating all treated patients as metabolically equivalent [2,49,50,51].

Available metabolomic studies suggest that some metabolites shift toward a control-like profile after GH treatment, whereas others remain altered, consistent with a mixture of GH-responsive and persistent GH-independent signatures. This distinction matters for biomarker interpretation: a treatment-responsive metabolite may serve as a pharmacodynamic readout of restored GH action, while a persistent abnormality may reflect sex, developmental stage, adiposity, disease history, incomplete tissue responsiveness, or metabolic pathways not directly corrected by GH exposure. Treatment-response studies should therefore distinguish normalization, partial correction, persistence, and new treatment-associated shifts rather than treating all post-treatment changes as evidence of restored physiology [9,47].

Treatment formulation is likely to become increasingly important in metabolomic studies of GHD. Daily recombinant human GH improves growth outcomes in children and body composition-related endpoints in adults, but it does not reproduce the temporal organization of endogenous GH secretion. Long-acting GH preparations add further complexity because they extend GH exposure or GH action across several days, producing pharmacokinetic and pharmacodynamic profiles that differ from both daily injections and physiological pulsatile secretion. These differences may influence downstream metabolic pathways, including IGF-1 production, lipid mobilization, carbohydrate handling, and substrate oxidation. Therefore, metabolomic and lipidomic studies should record and analyze GH formulation, dose, timing of sample collection relative to injection, treatment duration, adherence, and IGF-1 response. Without these variables, treatment-associated metabolic signatures may be difficult to distinguish from differences in drug exposure, sampling time, or incomplete tissue responsiveness [49,51,52].

## 6. Translational Relevance of Metabolomic and Lipidomic Biomarkers

The translational value of metabolomic and lipidomic signatures depends on biomarker purpose. In this review, we distinguish diagnostic biomarkers (classification of GHD), biological phenotype markers (characterization of downstream metabolic state), pharmacodynamic biomarkers (evidence of biological response to GH), predictive biomarkers (prediction of subsequent treatment response), and metabolic-risk biomarkers (identification of adverse metabolic phenotypes). A signature that is unsuitable for diagnosis may still be informative for treatment monitoring or response prediction. Current evidence remains investigational because studies are generally small, methodologically heterogeneous, and insufficiently replicated [9,12,53].

**Figure 3:**
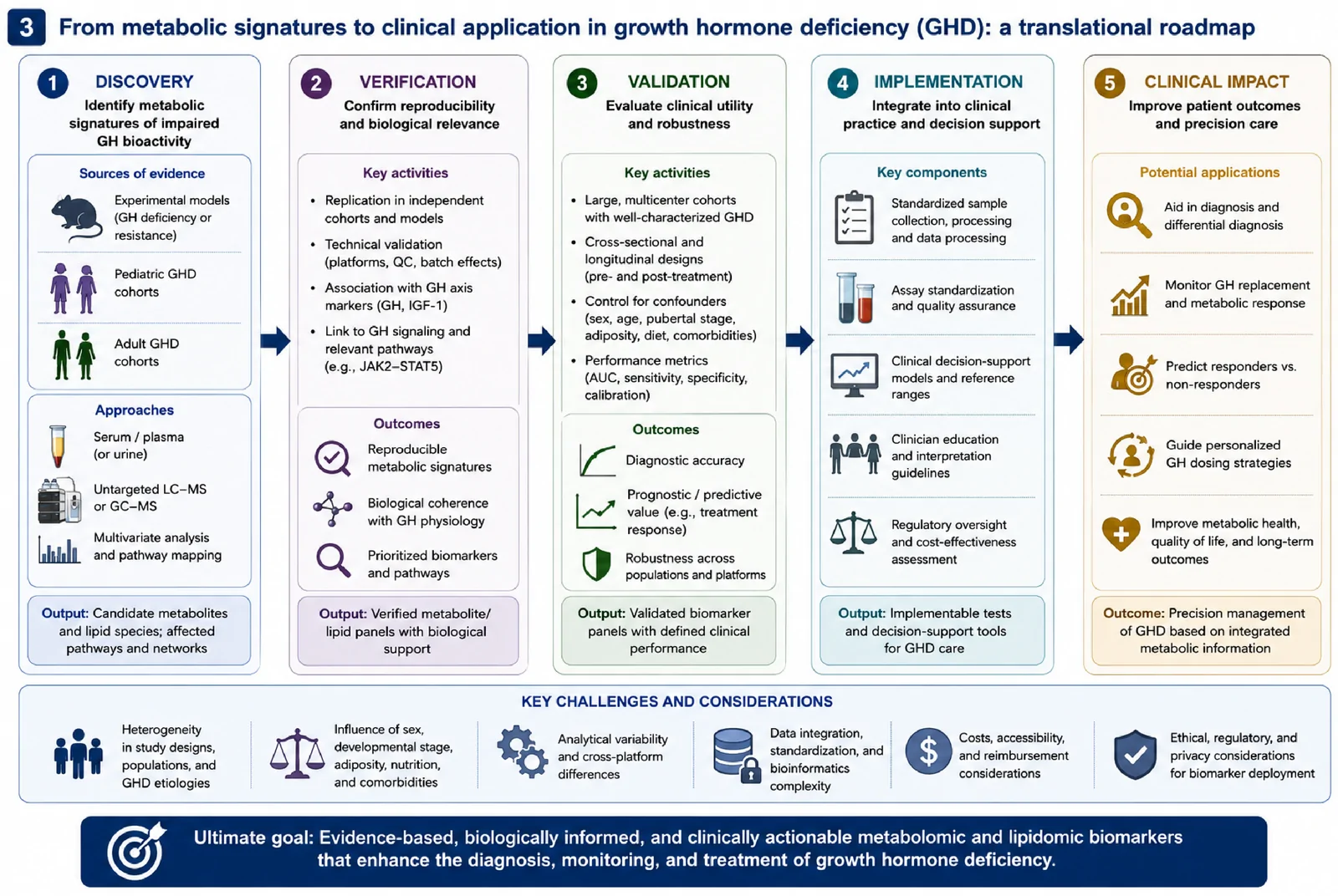
Translational roadmap from metabolic signatures to clinical application in growth hormone deficiency. This schematic places the reviewed evidence within a staged biomarker-development framework: discovery, analytical verification, biological and clinical validation, implementation, and clinical impact. Potential uses include diagnostic phenotyping, pharmacodynamic monitoring, prediction of treatment response, and metabolic-risk assessment, but all remain investigational. Standardization, external validation, clinically meaningful performance thresholds, feasibility, and regulatory qualification are required before routine clinical use. GH, growth hormone; IGF-1, insulin-like growth factor 1.

Metabolomic and lipidomic profiles may complement conventional endocrine assessment by capturing systemic consequences of altered GH action that are not apparent from static GH or IGF-1 measurements. Their most plausible near-term uses are biological phenotyping and longitudinal pharmacodynamic assessment, while diagnostic and predictive applications require stronger validation. Figure 3 summarizes the progression from discovery through analytical verification, biological and clinical validation, implementation, and eventual clinical impact. Importantly, these stages should not be conflated: analytical validation establishes assay performance and reproducibility, whereas clinical validation establishes performance in the intended patient population, and clinical utility requires evidence that biomarker use improves decision-making or outcomes [54,55,56].

Longitudinal assessment during GH replacement is a particularly plausible application. Serial sampling could identify pathways that normalize with treatment, remain persistently altered despite endocrine improvement, or respond differently according to formulation and exposure pattern. Such studies require standardized control of dose, adherence, treatment duration, fasting state, and sampling time relative to GH administration, particularly when daily and long-acting preparations are compared [4,9,51].

Integration of metabolomics with lipidomics, endocrine phenotyping, body-composition measures, insulin sensitivity, liver function, and clinical outcomes will be necessary to determine whether candidate signatures provide information beyond established assessment. Biomarker instability across cohorts can arise from biological heterogeneity, pre-analytical conditions, platform differences, preprocessing, and model overfitting; accordingly, longitudinal sampling, sex-stratified analyses, standardized analytical workflows, transparent preprocessing, and independent external validation should be prioritized [54,55,56,57]. These recommendations are intended to move candidate signatures from discovery associations toward reproducible and clinically interpretable biomarkers.

### 6.1. Diagnostic Stratification

Diagnostic stratification is attractive but remains one of the least validated applications of metabolomic and lipidomic profiling in GHD. Patients differ in developmental stage, adiposity, pituitary disease context, treatment history, and severity of impaired GH bioactivity [1,5,6]. Candidate diagnostic signatures must therefore demonstrate that they add information beyond GH stimulation testing, IGF-1, auxology, body composition, and routine metabolic assessment, and they must be tested against clinically relevant comparison groups rather than healthy controls alone [6,12,30].

In practical terms, diagnostic stratification would require metabolomic and lipidomic signatures to distinguish GH-related metabolic disruption from common clinical mimics. Obesity, undernutrition, chronic inflammatory disease, liver dysfunction, delayed puberty, poorly controlled diabetes, and estrogen exposure can alter IGF-1 concentrations and circulating metabolites, creating overlap with GHD-associated profiles. A useful biomarker panel would therefore need to show added value beyond GH stimulation results, IGF-1, auxology, body composition, and routine metabolic testing. It would also need to perform against relevant comparison groups, including short stature without GHD, obesity-related low GH secretion, delayed puberty, chronic illness, liver disease, and other pituitary disorders. Without these comparisons, a metabolite panel may distinguish one discovery cohort from controls while failing to identify GH-specific biology.

For diagnostic use, statistical separation in a discovery cohort is insufficient. Candidate biomarkers require reproducibility across platforms and sampling conditions and clinically meaningful classification performance in independent cohorts. The pediatric studies by Xu et al. and Aggelaki et al. identified discriminatory metabolic patterns, but neither established externally validated ROC-AUC thresholds for GHD diagnosis [8,53]. Reported diagnostic performance should therefore be interpreted alongside cohort size, validation strategy, and multiple-testing control; until independently replicated thresholds are available, metabolomic and lipidomic profiles should remain investigational tools for biological stratification rather than stand-alone diagnostic tests [5,7,12].

### 6.2. Treatment Monitoring and Pharmacodynamic Readouts

Treatment monitoring is conceptually distinct from diagnosis because a metabolite need not discriminate GHD from controls to serve as a pharmacodynamic readout. Longitudinal profiling before and after GH replacement could identify signatures of restored GH action, incomplete metabolic correction, or persistent GH-independent abnormalities. This approach may be particularly useful when growth or IGF-1 responses are discordant with broader metabolic outcomes, although current evidence remains insufficient for routine clinical use [1,4,8,47].

Treatment-response metabolomics should distinguish normalization, partial correction, persistence, and new treatment-associated shifts. Some metabolites may move toward a control-like profile after GH replacement, whereas others persist despite improved IGF-1 or clinical response, suggesting residual effects of sex, developmental stage, adiposity, disease duration, or GH-independent regulation [9,47]. In a pediatric PEG-rhGH study, eight baseline metabolites discriminated higher from lower subsequent growth-response groups with reported ROC AUCs ranging from 0.720 to 0.794, but the analysis was exploratory and lacked independent validation [58]. These data illustrate why treatment-response biomarkers should be evaluated separately from diagnostic biomarkers and externally validated before clinical interpretation.

For metabolomic and lipidomic profiling to be useful in treatment monitoring, GH exposure must be carefully defined rather than treated as a simple treated-versus-untreated variable. Daily recombinant GH and long-acting GH formulations differ in pharmacokinetic and pharmacodynamic profiles, and neither fully reproduces endogenous pulsatile GH secretion. Sampling time relative to injection, dose, adherence, treatment duration, IGF-1 response, pubertal status, adiposity, and concurrent pituitary hormone replacement can all influence downstream metabolic signatures. These variables are especially important when comparing daily and long-acting preparations, because differences in metabolite or lipid profiles may reflect exposure pattern, sampling time, or tissue-specific responsiveness rather than superior or inferior biological efficacy. Future treatment-response studies should therefore standardize sampling conditions, report formulation and dose clearly, and analyze metabolomic changes alongside IGF-1, growth response, body composition, insulin sensitivity, and safety outcomes [49,52].

### 6.3. Biological Pathway Mapping and Clinical Integration

Beyond individual biomarker discovery, metabolomic and lipidomic profiling can map the biological pathways through which reduced GH bioactivity reshapes systemic metabolism. Pathway-level interpretation may be more robust than isolated metabolite hits, but it remains an analytical synthesis rather than direct evidence of interorgan flux. Figure 3 places this biological interpretation within a translational framework in which candidate signatures must progress through verification, validation, and implementation before clinical application [54,55,59].

Clinical integration requires metabolomic and lipidomic data to be interpreted alongside endocrine and physiological measures rather than in isolation. In GHD, signatures should be linked to GH stimulation results, IGF-1, auxology or adult phenotype, body composition, insulin sensitivity, lipid profiles, liver function, treatment exposure, and relevant pituitary hormone replacement. Analytical validation should establish assay accuracy, precision, reproducibility, and robustness; clinical validation should then test biomarker performance in the intended population; and clinical utility requires evidence that using the biomarker improves classification, monitoring, or patient management [49,55,56,59].

For clinical use, metabolomic and lipidomic findings will need to be reported in a way that supports interpretation rather than overwhelms it. A useful framework should move beyond lists of statistically altered metabolites and classify signatures by biological domain, direction of change, treatment responsiveness, and relationship to clinical phenotype. This would allow investigators to separate pathway signals that reflect reduced GH bioactivity from changes driven by developmental stage, adiposity, nutrition, liver function, medication exposure, or analytical variation. In practice, metabolomic outputs may be most useful when translated into pathway-level summaries that can be compared with endocrine testing, body composition, metabolic risk markers, and treatment outcomes. Such an approach would make biomarker development more biologically grounded and less dependent on single-metabolite findings that may not reproduce across cohorts. The immediate goal should therefore be clinical interpretability, not premature clinical deployment [54,55,57].

## 7. Challenges and Limitations

Metabolomic and lipidomic profiling provides a powerful framework for studying the downstream consequences of impaired GH bioactivity, but current evidence is constrained by biological heterogeneity, pre-analytical and analytical variability, small and heterogeneous cohorts, limited longitudinal validation, and the narrative nature of the available evidence synthesis. Several of these limitations overlap; the key issues are summarized below to avoid repeating the same caveats across sections.

### 7.1. Biological Heterogeneity

Biological heterogeneity is a major barrier to reliable biomarker development. Age and developmental stage, sex, adiposity and lean mass, nutritional state, physical activity, liver function, insulin sensitivity, associated pituitary deficiencies, and GH treatment can all modify circulating metabolites and lipids [1,4,5,6,60]. Body composition is particularly important because increased visceral adiposity and reduced lean mass can mediate metabolic changes through altered insulin sensitivity, lipid flux, and amino-acid turnover. Thus, an observed GHD-associated signature may reflect a mixture of direct GH-dependent regulation and downstream consequences of the GHD phenotype.

Sex, developmental stage, and treatment exposure should therefore be treated as core design variables. Mixed-sex analyses can dilute sexually dimorphic responses; combining pediatric and adult cohorts can obscure developmental effects; and pooling treated and untreated patients can confound disease and pharmacodynamic signals [11,18,19,50].

### 7.2. Technical and Analytical Variability

Pre-analytical and analytical variability also limits cross-study comparability. Fasting duration, circadian sampling time, recent food intake and exercise, specimen type, processing delay, storage, medication exposure, and timing relative to GH administration can alter circulating profiles. Platform choice (NMR, GC-MS, LC-MS, targeted or untargeted approaches) determines metabolite coverage and annotation confidence, while batch correction, normalization, missing-value handling, and statistical thresholds can materially change the set of reported features [61,62,63,64]. Standardized collection protocols and transparent reporting are therefore essential.

Untargeted studies additionally face challenges of feature identification and multiple testing. Annotation confidence depends on mass accuracy, retention time, MS/MS quality, database matching, and consistent nomenclature; statistical results depend on preprocessing, scaling, correction for multiple comparisons, and feature-selection thresholds [63,65,66,67]. These details should be reported sufficiently to distinguish robust biological signals from technically or statistically driven findings.

### 7.3. Study Design Limitations

Most GHD metabolomic and lipidomic studies remain exploratory, cross-sectional, or based on modest sample sizes. This limits statistical power, restricts sex- or puberty-stratified analysis, and increases the risk of cohort-specific findings. The heterogeneity of populations, models, platforms, and endpoints also makes formal quantitative pooling inappropriate for the present review. No formal risk-of-bias scoring or evidence grading was performed; these are limitations of the narrative synthesis and should be considered when interpreting pathway-level convergence. Because the authors are active investigators in GH and GHD research, selection and interpretive bias cannot be completely excluded despite the structured search strategy, explicit evidence hierarchy, and reference verification.

### 7.4. Translational Barriers

Discovery-level findings do not automatically translate into clinically useful biomarkers. Candidate signatures must be analytically validated, externally replicated, clinically validated in the intended population, and shown to add value beyond established endocrine and clinical measures. Diagnostic, pharmacodynamic, predictive, and metabolic-risk applications should be evaluated separately because their required populations, endpoints, and performance thresholds differ [54,55,56].

Validated decision thresholds and reference frameworks are not yet available for GHD metabolomic signatures. Age, sex, developmental stage, adiposity, fasting state, liver function, medication exposure, treatment duration, and GH formulation can all shift biomarker distributions. These sources of variation will need to be incorporated into reference ranges or multivariable models before clinical interpretation is realistic.

Clinical implementation will also require practical and regulatory feasibility. Assays must be reproducible, scalable, cost-conscious, and compatible with clinically reasonable turnaround times; proposed panels will require standardized methods, external replication, prospective evaluation, and appropriate regulatory qualification before routine use. These requirements reinforce the current role of metabolomics and lipidomics as research and pathway-mapping tools rather than stand-alone clinical tests.

## 8. Future Directions

Future work should move from expanding discovery lists toward testing reproducible pathway signatures in clearly defined clinical contexts. Priorities include independent replication, standardized analytical workflows, predefined biomarker purposes, and explicit separation of direct GHD evidence from mechanistic models. The objective should be to identify signals that are robust, treatment-responsive where appropriate, and capable of adding information beyond established endocrine and clinical measures.

Longitudinal studies will be essential for determining whether metabolomic and lipidomic signatures reflect stable features of GHD, dynamic responses to GH replacement, or residual abnormalities that persist despite treatment. Cross-sectional comparisons can identify candidate pathways, but they cannot establish whether altered metabolites precede treatment, normalize with therapy, or remain resistant to correction. Serial sampling before and after GH initiation should therefore be linked to treatment duration, formulation, dose exposure, adherence, and timing of blood collection relative to injection. This will be especially important as long-acting GH preparations become more widely used, because different exposure profiles may produce distinct downstream metabolic effects. Linking longitudinal metabolomic changes to growth response, IGF-1, body composition, insulin sensitivity, lipid profiles, liver markers, and patient-relevant outcomes will be necessary to determine whether these signatures add meaningful biological or clinical information beyond conventional monitoring [9,47,49,50,51,52].

A stronger translational framework will require integration of complementary biological layers. Metabolomics and lipidomics provide functional readouts of small-molecule and lipid states closest to phenotype; transcriptomics identifies gene-expression programs that may generate or respond to these changes; and proteomics provides information on protein abundance, processing, and pathway activity. Integrating these layers with GH stimulation results, IGF-1, body composition, insulin sensitivity, liver function, lipid profiles, and clinical outcomes may help determine whether endocrine disruption, tissue-level regulation, and circulating metabolic phenotype converge on the same mechanism.

Mechanistic validation will be necessary to determine whether candidate metabolomic and lipidomic signatures reflect direct consequences of impaired GH signaling or secondary effects of altered growth, adiposity, nutrition, or treatment exposure. Animal models and tissue-specific approaches can help test whether changes in circulating amino acids, lipid species, or energy-related metabolites arise from altered GH action in specific tissues or from coordinated interorgan substrate exchange. These studies should be designed with sex as a core biological variable, because GH secretion, hepatic signaling, body composition, and metabolic response differ between males and females. Developmental timing also requires attention, particularly in pediatric GHD, where growth and puberty can reshape the same pathways affected by reduced GH bioactivity. The most useful future studies will combine controlled mechanistic experiments with carefully stratified human cohorts, allowing candidate signatures to be tested for biological origin, treatment responsiveness, and clinical relevance rather than simply reported as statistical associations.

## 9. Conclusions

GHD is not only a disorder of impaired growth or reduced circulating GH activity; it is a systemic endocrine–metabolic condition that alters substrate handling across GH-responsive tissues. Evidence from animal models and human studies suggests that impaired GH bioactivity is associated with coordinated changes in amino-acid turnover, mitochondrial substrate use, and lipid remodeling. These findings support the concept that metabolomic and lipidomic profiling can provide functional insight into downstream metabolic consequences of altered GH signaling that are not fully captured by GH stimulation testing or serum IGF-1 alone.

At present, metabolomic and lipidomic signatures in GHD should be viewed as promising biological readouts rather than validated clinical biomarkers. Existing studies remain limited by modest cohort sizes, platform variability, incomplete longitudinal validation, and biological heterogeneity. A pathway-based interpretation is currently the most defensible because recurrence is stronger across metabolic domains than across individual metabolites; however, this also reflects the present resolution and heterogeneity of the evidence. Improved analytical resolution, deeper phenotyping, and spatial or tissue-resolved approaches may ultimately reveal biologically distinct GHD subtypes or more specific metabolite signatures.

Future progress will depend on standardized analytical pipelines, longitudinal treatment-response studies, sex- and development-aware designs, and integration with endocrine, metabolic, and clinical phenotyping. With this level of validation, metabolomic and lipidomic profiling may help refine biological stratification, improve assessment of GH treatment response, and support the development of more physiologically informative biomarkers for GHD. Until then, the field should resist overinterpreting discovery signatures and focus on identifying metabolic signals that are reproducible, mechanistically grounded, and clinically useful. The primary metabolomic and lipidomic studies underpinning this review are summarized in Table 1.

## Figures and Tables

**Table 1:** Primary metabolomic and lipidomic studies relevant to growth hormone deficiency and altered GH bioactivity. The table summarizes population/model, analytical platform, study design and GH exposure, principal findings, and major interpretive limitations. Evidence is explicitly classified by proximity to classical GHD, distinguishing direct human GHD studies from GHD-enriched human cohorts, experimental models of reduced GH secretion, and mechanistic GH-resistance or tissue-specific GHR models. Platform type and multiple-testing procedures are reported where available. The table is intended to support comparative interpretation of the evidence rather than imply equivalent clinical weight across study types.

Study	Population/Model	Sample and Platform	Design/GH Exposure	Principal Findings	Major Interpretive Limitation
**A. Experimental models**					
Al-Samerria et al. [9]	Experimental GHD|Mouse; combined GHD (POU1F1/Pit-1 K216E); both sexes	Serum; untargeted LC–MS	GHD mutant and WT comparators with GH-treatment assessment.	GHD- and GH-responsive signatures involving energy, purine and amino-acid metabolism; marked sex dependence.	Combined pituitary phenotype; circulating signatures do not establish tissue origin and require independent validation.
Hoffman et al. [38]	Experimental reduced GH secretion|Mouse; GHRH-knockout; male and female	Serum global metabolomics plus BAT transcriptomics; metabolomics *n* = 3/sex/genotype	Cross-sectional genotype-by-sex comparison.	Mitochondrial and amino-acid pathways were altered; strong genotype-by-sex interactions.	Very small metabolomics groups; longevity model and integrated exploratory analysis limit direct clinical extrapolation.
Riedel et al. [39]	Mechanistic GH resistance|Pig; global GHR deficiency; 4 males and 4 females/group	Liver; targeted metabolomics integrated with shotgun proteomics	GHR-deficient versus GHR-expressing controls.	Amino-acid degradation, urea-cycle and TCA-cycle remodeling; acylcarnitine pattern suggested reduced mitochondrial fatty-acid import/β-oxidation.	Models GH resistance rather than pituitary GHD; tissue-based targeted profiling is not a circulating biomarker study.
Young et al. [33]	Mechanistic mixed GH-axis models|Mouse; bGH transgenic, GHR-knockout and GH-knockout models; both sexes where applicable	Plasma/feces targeted amino-acid profiling; liver gene expression; acute serum glycine assay	Models spanning GH excess, GH resistance and acute GH replacement.	GH action changed circulating glycine and hydroxyproline in opposing directions; hepatic SHMT regulation provided mechanistic support.	Focused amino-acid study rather than broad metabolomics; heterogeneous GH-axis models limit generalization to GHD.
Yu et al. [68]	Mechanistic tissue-specific GH resistance|Mouse; hepatocyte-specific GHR deletion	Bile-acid profiling, liver assays and gut–microbiome analysis	Hepatic GHR deletion versus controls.	Altered bile-acid composition and microbiota, implicating hepatic GHR/CYP8B1-dependent regulation.	Mechanistic bile-acid study rather than a diagnostic metabolomics study; tissue-specific GH resistance is not pituitary GHD.
**B. Human studies**					
Abd Rahman et al. [47]	Direct human GHD|Female adolescent with severe GHD due to PIT-1 mutation; *n* = 1	Urine; 1H-NMR metabonomics	Longitudinal before and during 12 months of GH replacement.	GH therapy produced changes in urinary metabolites related to energy and muscle metabolism, supporting dynamic metabolic responsiveness.	Single-patient case; combined pituitary defect and urinary matrix preclude generalization.
Xu et al. [53]	Human GHD-enriched short-stature cohort|Children with short stature, predominantly related to endocrine disorders/GHD (*n* = 45), versus controls (*n* = 35)	Serum; NMR-based metabolomics	Cross-sectional	Higher citrate, phenylalanine, creatinine and tyrosine; lower glucose, serine, betaine, inositol, lysine, glycerol and glutamine.	Short-stature cohort was not a clean isolated-GHD population; cross-sectional design and no external validation.
Höybye et al. [30]	Direct human adult GHD|Adults with severe GHD (*n* = 10) versus age- and sex-matched controls (*n* = 10)	Fasting morning serum; GC–MS; 285 measured metabolites	Case–control plus 6 months of GH replacement.	Metabolic profile distinguished GHD from controls; 13 metabolites contributed most to separation; selected metabolites changed with GH but the overall profile remained distinct.	Small heterogeneous cohort; 11/13 leading discriminatory compounds were unidentified; no external validation.
Li et al. [58]	Direct human pediatric GHD, predictive-treatment study|Children with GHD treated with PEG-rhGH (*n* = 57)	Baseline serum; GC–TOF–MS metabolomics	High-response (*n* = 28) versus lower-response (*n* = 29) groups after treatment.	Baseline fatty-acid- and energy-related metabolites differed by subsequent growth response.	Post hoc response grouping, no untreated/control cohort and no independent validation; predictive claims remain exploratory.
Yang et al. [48]	Direct human adult GHD|Adults with GHD (*n* = 31) versus controls (*n* = 31)	Serum; untargeted UPLC–MS metabolomics	Cross-sectional; 11 patients followed during 12 months of rhGH.	Unsaturated-fatty-acid, sphingolipid and glycerophospholipid pathways were perturbed; selected lipid-related metabolites changed during rhGH.	Only 11 longitudinal participants; single-center cohort; treatment did not establish a universal normalized signature.
Zhang et al. [37]	Human hypopituitarism/GHD-associated fatty liver plus experimental model|Adults with hypopituitarism/GHD-associated fatty liver disease; complementary hypophysectomized rat model	Human serum untargeted metabolomics; rat liver proteomic/phosphoproteomic analyses	Human disease comparison plus experimental rhGH intervention.	Linked impaired GH action with oxidative stress, mitochondrial and lipid-related pathways; experimental work supported hepatic mechanisms.	Complex hypopituitarism and liver disease limit attribution specifically to isolated GHD; mechanistic findings rely partly on the rat model.
Yang et al. [46]	Direct human adult GHD|Adults with AGHD after intracranial germ-cell tumors (*n* = 30) versus age-, sex- and BMI-matched controls (*n* = 30)	Fasting serum; high-coverage targeted lipidomics; 534 lipids from 29 classes	Cross-sectional GHD versus matched controls.	Broad elevations in several neutral and membrane-lipid subclasses with lower ether-linked PE/PC species; lipid patterns correlated with adverse metabolic traits.	Etiologically restricted cohort; targeted lipid associations require replication in broader AGHD populations
Aggelaki et al. [8]	Direct human pediatric GHD|Children with confirmed GHD (*n* = 22) and controls (*n* = 48); treated subset *n* = 15	Fasting plasma, serum and urine; untargeted/targeted 1H-NMR	Cross-sectional plus paired 3-month GH follow-up; FDR correction applied to univariate metabolite testing.	Distinct baseline metabolic fingerprint with ketone-body and amino-acid differences; post-treatment changes were subtle with limited discrimination.	Small treated subset, short follow-up and no independent validation.
Shilo et al. [12]	Direct human pediatric GHD diagnostic cohort|Children undergoing GH stimulation testing; GHD *n* = 25, controls *n* = 41	Serum; untargeted LC–MS; 951 metabolites (280 polar, 671 lipids)	Prospective diagnostic cohort; FDR correction reported for individual-metabolite testing.	Seven polar metabolites and 50 lipids differed nominally; only PS (40:3) survived FDR correction; two lipid clusters were associated with GHD.	Modest cohort; limited FDR-significant individual markers; diagnostic thresholds influenced separation and independent validation is lacking.

AGHD, adult growth hormone deficiency; BAT, brown adipose tissue; bGH, bovine growth hormone; BMI, body mass index; FDR, false discovery rate; GC–MS, gas chromatography–mass spectrometry; GC–TOF–MS, gas chromatography–time-of-flight mass spectrometry; GH, growth hormone; GHD, growth hormone deficiency; GHR, growth hormone receptor; GHRH, growth hormone-releasing hormone; LC–MS, liquid chromatography–mass spectrometry; NMR, nuclear magnetic resonance; PEG-rhGH, pegylated recombinant human growth hormone; PC, phosphatidylcholine; PE, phosphatidylethanolamine; PS, phosphatidylserine; rhGH, recombinant human growth hormone; SHMT, serine hydroxymethyltransferase; TCA, tricarboxylic acid; UPLC–MS, ultra-performance liquid chromatography–mass spectrometry. Bold text denotes study-category headings.

## Data Availability

No new data were created or analyzed in this review. Data sharing is not applicable to this article.
